# Exposure to polystyrene microplastic beads causes sex-specific toxic effects in the model insect *Drosophila melanogaster*

**DOI:** 10.1038/s41598-022-27284-7

**Published:** 2023-01-05

**Authors:** Samar El Kholy, Yahya Al Naggar

**Affiliations:** Zoology Department, Faculty of Science, Tanta University, Tanta, 31527 Egypt

**Keywords:** Zoology, Environmental sciences

## Abstract

The toxicity of MPs on aquatic creatures has been extensively studied, but little attention was paid to terrestrial organisms. To fill this gab, we conducted a series of experiments using *Drosophila* as a model organism to understand whether exposure to different concentrations (0.005, 0.05, 0.5 µg/ml) of polystyrene microplastics (PS-MPs) beads (2 µm in size) can impact flies feeding activity, digestion and excretion. The ability of flies to distinguish between normal and PS-MPs treated food media was tested first, and then we evaluated the effects of a 7-day short-term exposure to PS-MPs on food intake, mortality, starvation resistance, fecal pellet count, and the cellular structure of mid gut cells. The results revealed that flies can really differentiate and ignore MPs-treated food. We discovered sex-specific effects, with male flies being more sensitive to PS-MPs, with all males dying after 14 days when exposed to 0.5 µg/ml of PS-MPs, whereas female flies survived more. All male flies exposed to PS-MPs died after 24 h of starvation. Midgut cells showed concentration-dependent necrosis and apoptosis in response to PS-MPs. Our findings provide new insights into MPs toxicity on terrestrial organisms and giving a warning that management measures against MPs emission must be taken.

## Introduction

Over the past few years, emerging pollutants known as microplastics (MPs) have received a lot of attention^1–3^. Recent studies have shown that MPs can be found in the air, soil, and water, among other environmental matrices^4^. Humans may consume up to 52,000 particles annually, but estimates of inhaled MPs—which may be as high as 74,000 particles annually—are even more worrisome^5^.

Due to their capacity to assemble organic contaminants, MPs, which are plastic particles with a diameter of less than 5 mm, are a cause for concern^6^. Depending on where they came from, they can be separated into two categories primary MPs and secondary MPs. Those in the major subgroup are frequently found in consumer goods like cosmetics, detergents, and cleaning supplies and are produced directly as tiny materials. In dockyards, primary MPs are frequently used as blast cleaning agents for heavy materials like ship hulls. Secondary MPs are created when larger plastic materials deteriorate (break down) as a result of natural weathering processes in the air or in water. For instance, municipal sewage sludge, road wear particles, and tires have all been mentioned as potential sources of microplastics in the environment^7,8^. Due to the significant amount of plastic that enters the environment, it is believed that the majority of environmental MPs are secondary MPs^9,10^.

Because of the widespread presence of MPs contamination, ecotoxicologists are concerned about their potential toxicity^11^. MP exposure can have toxicological effects on aquatic biota, such as decreased fitness, increased oxidative stress, immunological reactions, and impaired intestinal function^12,13^ as well as mammal lung inflammation^14,15^. Additionally, there is evidence that MPs interact with terrestrial organisms, including invertebrates, terrestrial fungi, and animal pollinators, that mediate crucial ecosystem services and functions^16–24^.

Despite extensive research on the toxicity of micro- and nano-plastics in aquatic life, studies in terrestrial systems are much less common^25^. As a result, there is a pressing need to bridge this knowledge gap to gain a better understanding of the effects of plastic pollution on terrestrial organisms. *Drosophila* is an ideal terrestrial model for research due to its short lifespan, simple and inexpensive maintenance, and the fact that the *Drosophila* genome has been fully sequenced^26,27^, more than 75% of human disease related genes have fly homologs. Consequently, we used *Drosophila melanogaster* as a model to study the effects of short-term exposure to different concentrations of PS-MPs beads as previously used in various toxicological studies^28–31^. Previous research revealed that exposure to PS micro- and nano-plastics affect locomotion, daily activity, caused significant morphological defects and genotoxicity of the flies^32–34^. Long-term exposure to polyethylene terephthalate (PET) MPs also had sex-specific effects on the flies' lifespan^35^ resulting in decreased egg production in female flies and decreased lipid, glucose content and starvation resistance in male flies^36^. Additionally, the effects of polyethylene and polyvinyl chloride exposure, which may or may not contain chemical additives, on the life history traits and immune response of *D. melanogaster* were investigated^37^. The life cycles of the offspring of treated flies raised in non-supplemented food were shorter, and the sex ratio and fertility of the flies treated with plastics in food media changed^37^. It is still unclear, though, whether flies can distinguish between food containing MPs and food without, as well as whether MPs can affect feeding behavior and regulate flies' excretion and digestion. These insights are critical for a better understanding of MP toxicity in living organisms.

The aim of this study was to evaluate the effects of short-term PS-MP exposure on the fruit fly *D. melanogaster*. To do so, we measured a variety of lethal and sublethal endpoints such as mortality, food choice, food intake, starvation resistance, fecal pellet count, and potential effects on the cellular structure of mid gut cells. We hypothesized that exposure to PS-MPs beads would negatively affect *Drosophila* flies by influencing feeding activity and modulating digestion and excretion, which could be concentration or/and sex dependent.

## Results

### Effect of PS-MPs on food choice

**Figure 1:**
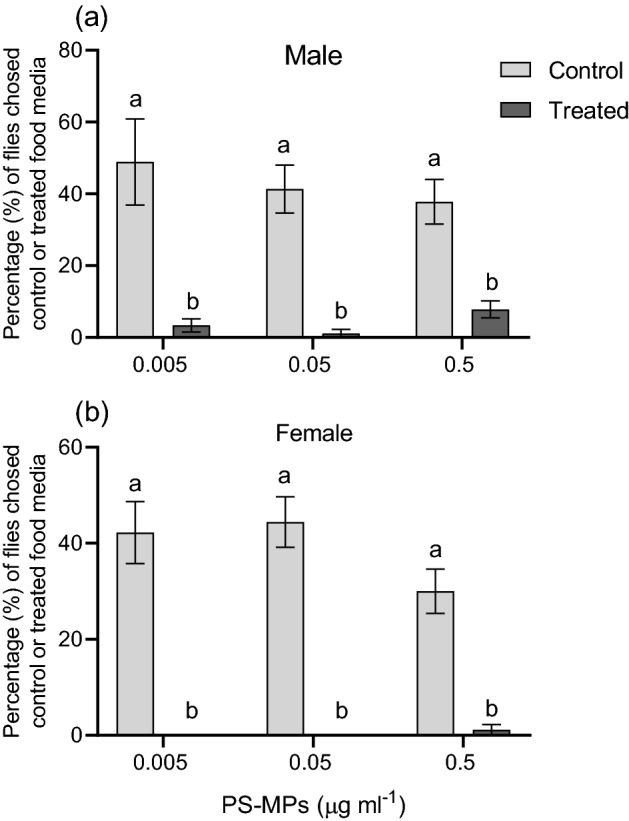
Percentage (%) (mean ± standard error mean (SEM)) of (**a**) male and (**b**) female flies (n = 10) chose control or food media treated with different concentrations of polystyrene microplastics (PS-MPs). Flies were free to move around and choose their preferred food before being counted every 5 min for 30 min as they stood and attempted to feed on each food. Flies were able to discriminate between control and PS-MPs treated food media regardless of the concentrations of PS-MPs and chased non-treated food media. Different lower-case letters denote a significant difference between treatments (student *t*-test, *P* < 0.05).

Flies were allowed to move around and select their preferred food before being counted every 5 min for 30 min while standing and attempting to feed on each food. All male and female flies were able to distinguish between control and PS-MPs-treated food media regardless of PS-MPs concentrations and chased non-treated food media except only 7% of male flies chasing the greater PS-MPs concentration (Fig. 1).

### Effect of PS-MPs on food intake

**Figure 2:**
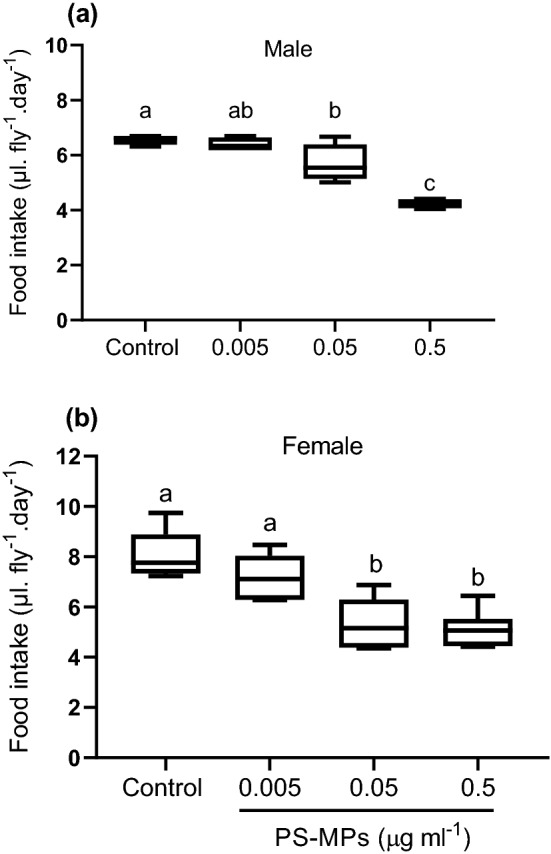
Food intake (μl. day^−1^. fly^−1^) of adult (**a**) male and (**b**) female flies (n = 9) fed sucrose solution (5% w/v) mixed with different PS-MPs (0.5, 0.05, 0.005 µg/ml) compared to flies fed non-treated (control) sucrose solution. Different lowercase letters indicate statistical differences between treatments (One way ANOVA, *p* < 0.05).

Male and female flies fed on sucrose solution spiked with either 0.05 or 0. 5 µg/ml of PS-MPs significantly consumed less food (male: F = 29.64, df = 3, *p* < 0.001, female: F = 15.21, df = 3, *p* < 0.001) compared to flies fed on non-treated sucrose solution (control) and flies fed on lesser concentration of PS-MPs (0.005 µg/ml) (Fig. 2). While male and female flies fed on sucrose solution that contained lesser PS-MPs concentration showed no discernible difference (*P* > 0.05) in their food consumption compared to control (Fig. 2).

### Effect of PS-MPs on survival

**Figure 3:**
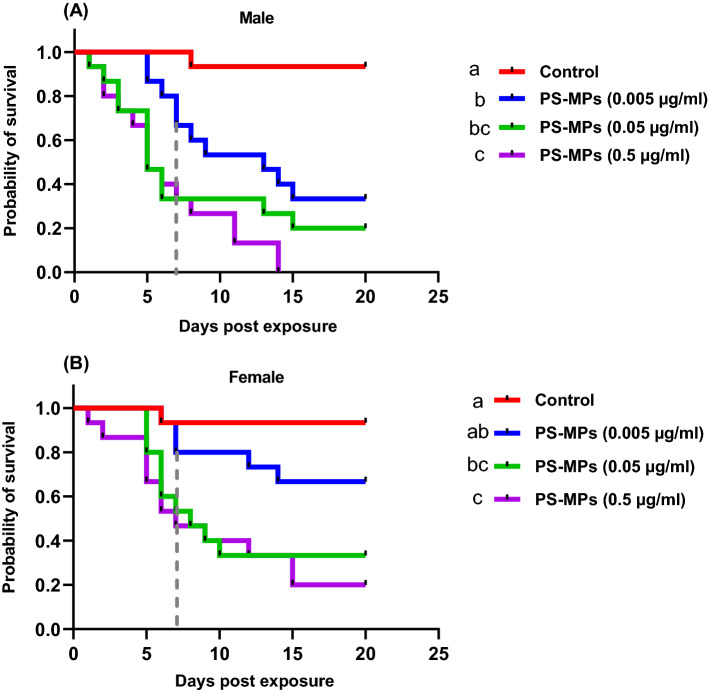
Kaplan–Meier survival curves showing effects of different concentrations polystyrene microplastics (PS-MPs) on survival of newly emerged male (**A**) and female (**B**) adult *D. melanogaster* flies. Flies were exposed for 7 days to a control or food media spiked with 0.005 or 0.05 or 0. 5 µg/ml of PS-MPs and then transferred to normal cultural media every two days. Different lowercase letters indicate statistical differences between treatments after Bonferroni correction (log-rank (Mantel cox) paired test, *p* < 0.008). Vertical dashed line indicates the end of PS-MPs exposure time.

Male and female *D. melanogaster* fly survival was significantly lower than controls after 7 days of chronic exposure to food media spiked with various PS-MP concentrations (log-rank (Mantel cox) paired test, male: X^2^ = 31.98, df = 3, *p* < 0.008; female, X^2^ = 20.97, df = 3, *p* < 0.008 after Bonferroni correction). Except for the female flies exposed to 0.005 mg/ml PS-MPs, which showed a reduction in survival but was not significantly different, all tested PS-MPs concentrations significantly (*p* < 0.05) decreased both male and female fly survival. Male flies also showed more sensitivity to PS-MPs, with all males dying after 14 days when exposed to 0.5 µg/ml of PS-MPs, whereas 20% of female flies survived to day 20 (Fig. 3).

### Effect of PS-MPs on starvation resistance

**Figure 4:**
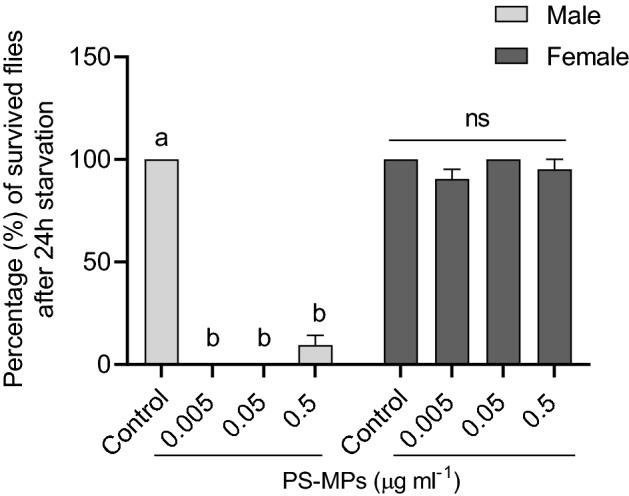
Percentage (%) (mean ± standard error mean (SEM)) of (**a**) male and (**b**) female flies (n = 7 pairs) survived after 24 h of starvation. Newly emerged flies (male and females) were exposed to different concentrations of polystyrene microplastics (PS-MPs) before being starved for 24 h. All female flies survived while almost all male flies died regardless of the concentration of PS-MPs. Different lowercase letters indicate statistical differences between treatments (One way ANOVA, *p* < 0.05). ns, indicate no significant differences.

An organism's ability to survive starvation may be a sign of its fitness. So, when we compared the starvation resistance for 24 h of newly emerged flies (male and females) exposed to different concentrations of PS-MPs for 7 days, we found that substantially all male flies exposed to either PS-MPs died, regardless of the concentration used. Surprisingly, almost all female flies survived and were resistance to starvation (Fig. 4).

### Effect of PS-MPs on defecation rate

**Figure 5:**
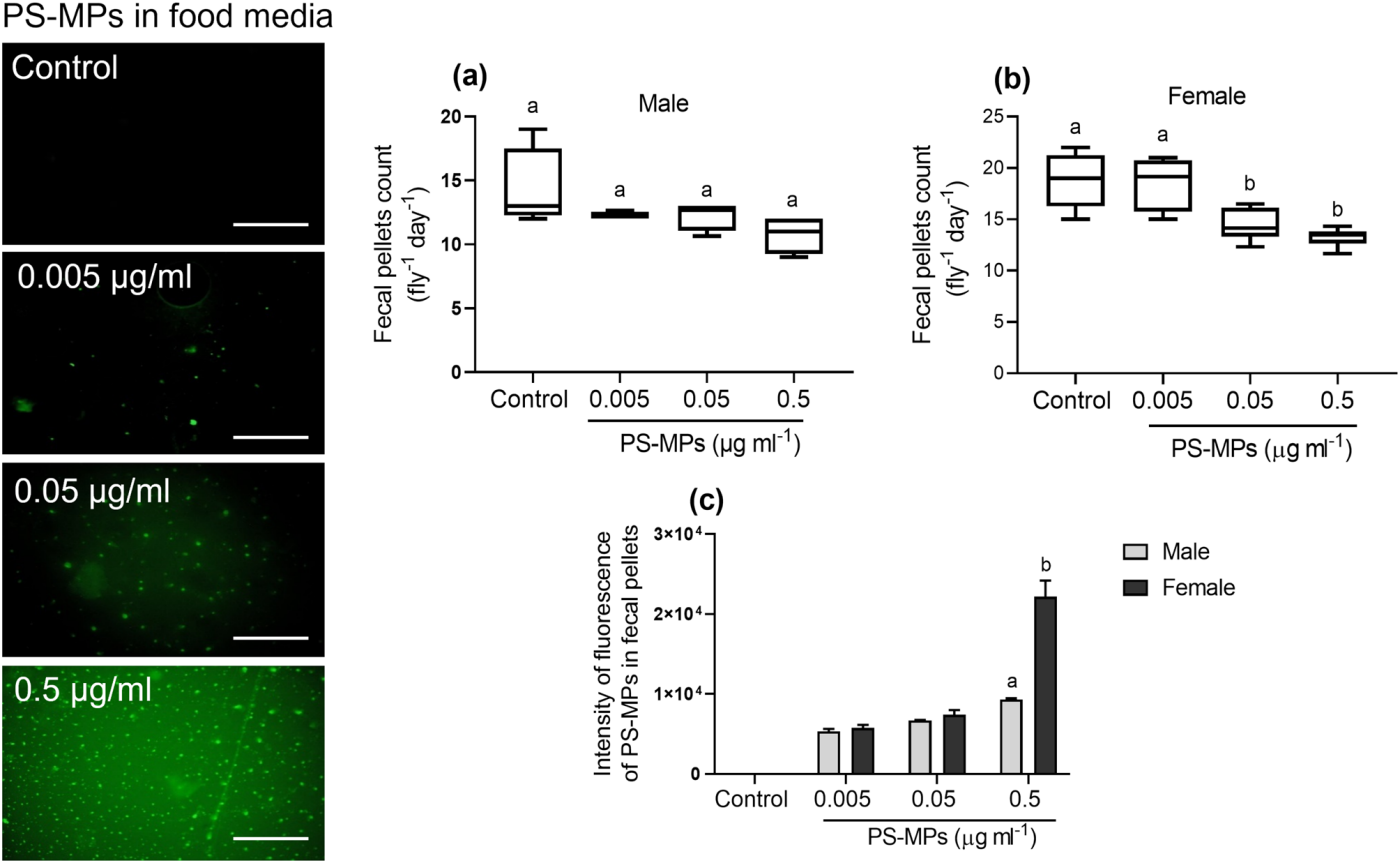
Showing polystyrene microplastics (PS-MPs) in food media (green dots) and the fecal pellets count of adult (**a**) male, (**b**) female flies (n = 5) fed food media either free of PS-MPs (control) or PS-MPs supplemented for 7-days and (**c**) integrated intensity of the fluorescence of PS-MPs in fecal pellets of both male and female flies. Symbols on the box plot represent maximum and minimum values (whiskers: ┬ ┴), mean values (-). Column bars (Mean ± SEM) and box plot with different lowercase letters denote significant differences among treatments (one-way ANOVA with Tukey post hoc test *P* < 0.05). Scale bar: 50 µm.

Fecal pellets of both male and female flies fed control or food media treated with different concentrations of PS-MPs for 7 days were counted to quantify the effect of PS-MPs on the defecation rate. Sex-specific discrepancy was observed where only female flies fed media spiked with 0.05 or 0.5 µg/ml of PS-MPs had substantially less fecal pellets than both the control female flies fed standard food media without PS-MPs and flies fed food media treated with 0.005 µg/ml of PS-MPs (F = 11.18 df = 3, *p* < 0.001). There was no significant difference (*p* > 0.05) in defecation rate of both female flies exposed to the lesser concentration of PS-MPs and male flies exposed to different concentrations of PS-MPs compared to control (Fig. 5a, b).

PS-MPs were found in fecal pellets of both male and female flies fed on food media treated with different concentrations of PS-MPs (Fig. S1). The integrated intensity of PS-MP fluorescence in fecal pellets of only female flies fed 0.5 µg/ml of PS-MPs-supplemented food media was significantly higher than that quantified in fecal pellets of males fed the same concentration (One-way ANOVA, *P* ˂ 0.05; Fig. 5c). Furthermore, adults excreted many fecal pellets that fell uncontrollably while walking, similar to those seen in patients suffering from diarrhea or urinary incontinence. Both males and females experienced this phenomenon (Fig. S1).

### Detection and distribution of PS-MPs in body tissues

**Figure 6:**
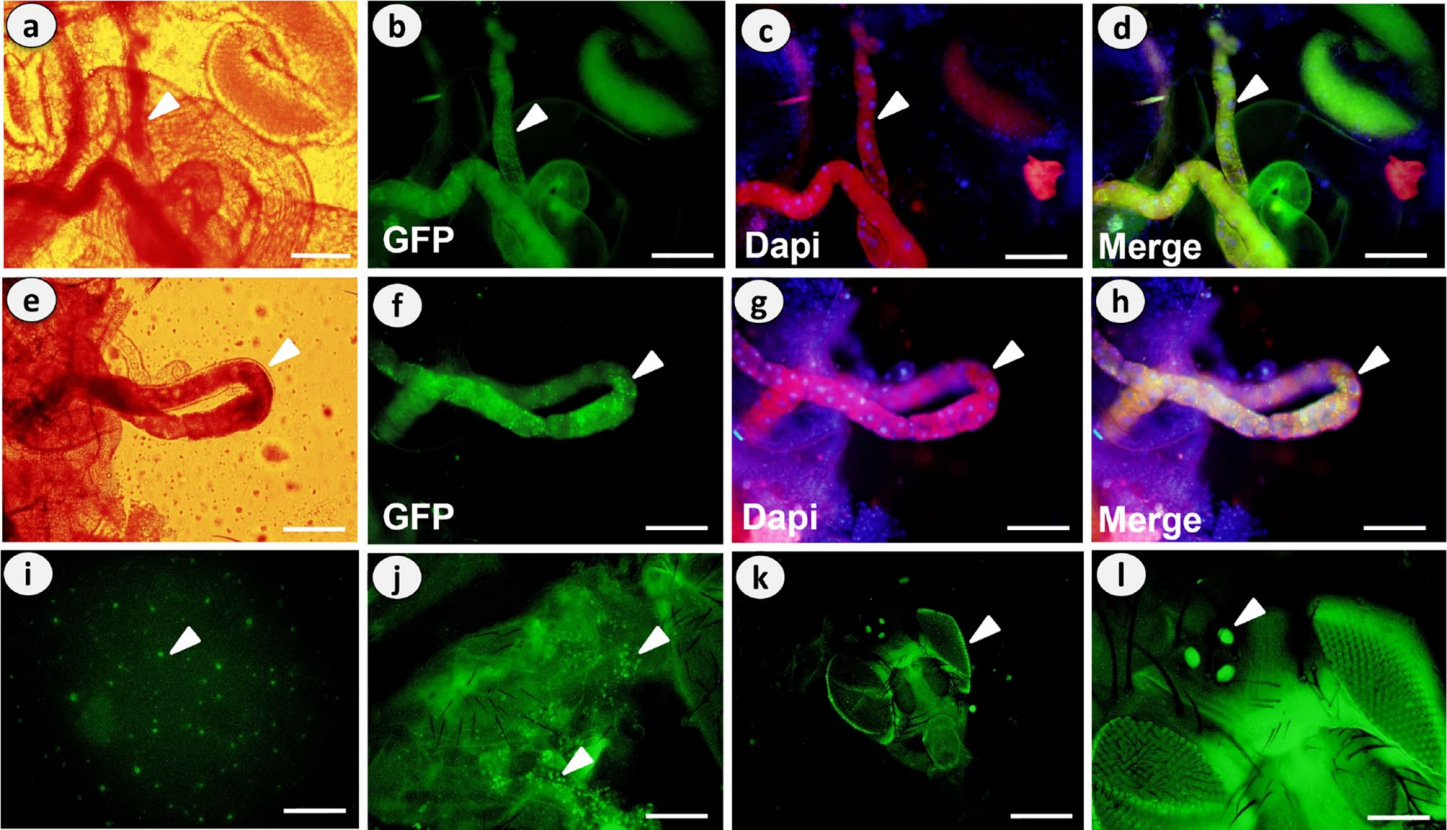
Showing the tissues of adult *D. melanogaster* flies that fed on food media-supplemented with different concentrations of green-yellow, fluorescent polystyrene microplastics (PS-MPs) for 7 days. Tissues were stained immunohistochemicaly using anti-GFP antibody (green florescent micrograph) and with DAPI fluorescent stain which turned the nuclei blue and the overlay of these two florescent micrographs revealed the presence and distribution of PS-MPs in the alimentary canal (**a**-**d**), and Malpighian tubules (**e**–**h**), hemolymph (**i**), body cavity (**j**) and in compound eyes and ocelli (**k** & **l**). Scale bar: 50 µm (**a**-**h**), 200 µm (**i**, **j** and **l**) and 100 µm (**k**).

We found PS-MPs in the gut, body cavity, Malpighian tubules, hemolymph, compound eye and ocelli of adult *D. melanogaster* flies (Fig. 6).

### Ultrastructural changes induced by MPs ingestion

**Figure 7:**
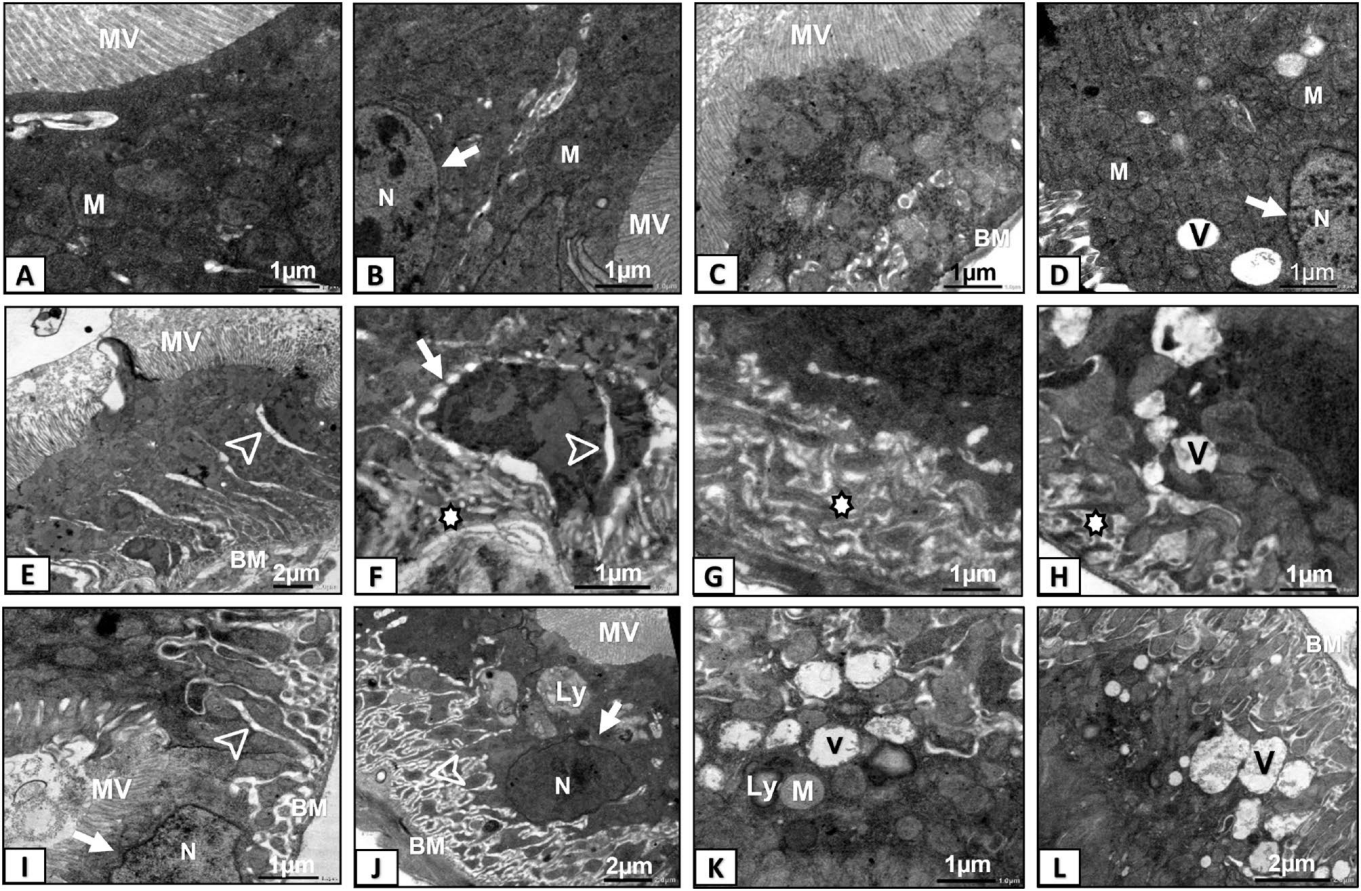
Transmission electron microscopy photomicrographs of mid gut cells of adult *D. melanogaster* flies that fed on food media-supplemented with different concentrations of green-yellow, fluorescent polystyrene microplastics (PS-MPs) for 7 days. (**A**, **B**) Control group, exhibiting typical morphology of columnar cells with the apical border straight and bearing numerous, long filament like microvilli. (**C**, **D**) flies fed 0.005 µg/ml of PS-MPs, showing comparable cellular structure like control group with only some vacuoles. E–H flies fed 0.05 µg/ml of PS-MPs. Note, intracellular cleft which also appeared within the nucleus (arrowhead), vacuolated cellular membrane (white arrow), abnormal folding of basement membrane (star) and vacuoles. I-L flies fed 0.5 µg/ml of PS-MPs. Note cracks or intercellular cleft, indentation in the nuclear membrane (white arrow), lysis of matrix and breakage of mitochondrial cristae, lysosomes engulf PS-MPs and large vacuoles N Nuclei; MV microvilli; M mitochondria; BM basement membrane; Ly lysosomes; V vacuoles.

The midgut epithelium of control group exhibited typical columnar cell morphology with the apical border, which was straight bearing multiple, long filaments like microvilli. Large and dense mitochondria and an oval nucleus were also found (Fig. 7A, B). Effects of PS-MPs on cellular structure of midgut cells were concentration-dependent. In flies fed 0.005 µg/ml of PS-MPs for 7 days, the ultrastructure of midgut cells was comparable to those of control group with only some vacuoles (Fig. 7C, D). Ultrastructure’s of midgut epithelial cells of flies exposed to 0.05 or 0.5 µg/ml of PS-MPs were adversely affected. Intracellular clefts were observed between columnar cells and within the nucleus, as well as vacuolated cellular membrane, abnormal basement membrane folding, and vacuoles in flies fed 0.05 µg/ml PS-MPs (Fig. 7E-H). Similarly, cracks or intercellular clefts, indentation in the nuclear membrane, matrix lysis and breakage of mitochondrial cristae, lysosomes engulfing PS-MPs, and large vacuoles were observed in flies fed 0.5 µg/ml PS-MPs (Fig. 7I-L).

## Discussion

We found that flies could actually distinguish between MPs-treated and untreated food, and that exposure to PS-MPs had toxic effects that were specific to sex, with male flies being more sensitive to PS-MPs and unable to survive after a 24-h starvation.

The presence of defensive or harmful compounds is one of the factors that influence what and how much animals consume^38,39^. In the current study, both male and female flies were able to distinguish and avoid PS-MPs-treated food regardless of PS-MPs concentrations. Moreover, flies fed on sucrose solution spiked with either 0.05 or 0. 5 µg/ml of PS-MPs significantly consumed less food compared to flies fed on non-treated sucrose solution (control). Similarly, *Gammarus pulex*, a freshwater shrimp, avoided a diet containing acrylic microfibers when given an alternative diet containing no microfibers^40^ and *Achatina fulica*, a terrestrial snail, consumed less food when fed on PET microplastic fibers^41^. This implies that MPs have a repellent effect, though the mechanism is unknown and needs further investigation. Additionally, the reduction in feeding activity may have long-term effects on the physiology, behavior, and fitness of the flies due to difficulties with the energy budget and the entire metabolism^11,42^.

In the present study, chronic exposure of *D. melanogaster* flies to food media spiked with various concentrations of PS-MPs for only 7 days resulted in significantly lower survival of both male and female flies beyond their exposure time in comparison to controls. A similar pattern was observed in nematode worms (*Caenorhabditis elegans*) after 3 days of exposure to 1.0 mg L^−1^ PS, but at the nanoscale^43^. In contrast, dialyzed PS spheres of different sizes and concentrations have low mortality rate on *D. melanogaster* flies^32,44^. Furthermore, at comparable dialyzed PS particle exposure concentrations, PS spheres have a low mortality rate in mice^45^ and different species of bacteria and algae^46,47^. It's interesting to note that the current study used commercial PS particle suspensions that hadn't been washed (dialyzed), but they also didn't contain sodium azide, a toxic preservative^48^. This implies that dialysis has an effect on MPs toxicity, which should be taken into account when assessing the ecological risk of MPs to living organisms.

Sex‐specific responses in *Drosophila* to some physiological, environmental and ecological stressors are common^35,49–51^ . Here, male flies were found to be more sensitive to PS-MPs, with all males dying after 14 days when exposed to 0.5 g/ml PS-MPs, whereas 20% of female flies survived to day 20, even though both sexes avoided sugar solution at a similar rate when spiked with this PS-MPs concentration. Furthermore, regardless of concentration, nearly all male flies exposed to PS-MPs died after 24 h of starvation, indicating a modulation in male flies' capacity and fitness to withstand starvation as a result of PS-MP exposure. While only female flies fed media containing 0.05 or 0.5 µg/ml PS-MPs had significantly fewer fecal pellets. In contrast, when male and female *D. melanogaster* flies were chronically exposed to PET-MPs, male flies lived longer than females^35^. These sex differences could be attributed to the type of plastic particle (PS vs. PET) or/and the fact that male and female flies respond differently to environmental intervention due to flies' dissimilar metabolic levels, which may contribute to sex differences in whole-body toxicity^52^. The latter is true because female dietary restricted (DR) flies lived up to 60% longer than starved or fully fed female flies, whereas male DR flies only lived up to 30% longer, indicating differences in nutrient/energy demand and allocation/utilization between sexes^53^.

The ability to survive starvation varies greatly across the animal kingdom, even among closely related species. Animals may develop resistance to starvation stress by reducing energy expenditure^54–56^. Furthermore, positive correlations were observed between starvation and desiccation tolerance in temperate *Drosophila* species^57^. In the current study, when we examined male flies died after 24 h starvation period, we found their carcasses were very desiccated. This implies that PS-MPs reduced the male flies' ability and fitness to resist starvation, which may have contributed to their desiccation and death after a 24-h period, most likely by increasing the rate of water loss. Our findings are consistent with previous research that found that plastic film contamination in soil increased the rate of soil water evaporation, resulting in desiccation cracking on the soil surface^58^. Sex-specific differences in *D. kikkawai* desiccation resistance have also been reported^49^, with female flies having higher desiccation resistance than males, which may be due to higher levels of body water, hemolymph, carbohydrates, and dehydration tolerance in females. The latter is true as female flies' fecal pellet counts considerably decreased in response to greater PS-MP concentrations in the current study, suggesting that female flies alter their physiological and metabolic states to cope with different stressors. More research is needed to gain a better understanding of the mechanisms underlying the observed desiccation caused by PS-MPs in male flies after 24 h starvation period.

MPs accumulation in tissues can have a variety of negative consequences^59,60^. We discovered fluorescent PS beads in the gut tissues, Malpighian tubules, hemolymph, and body cavity of adult *D. melanogaster* flies, which could cause disruptions in energy and lipid metabolism as well as oxidative stress, as previously reported^59^. Using a *Drosophila* larvae in vivo model, it was also possible to observe how nano-polystyrene plastics (PSNPLs) interact with gut lumen components, are taken up by gut enterocytes, cross the intestinal barrier to the hemolymph, and are taken up by hemocytes^61^. Previous research has also shown that PS-MPs can be ingested and accumulated in honey bee midgut and hindgut, as well as transported to the trachea and Malpighian tubules^62,63^. Interestingly, after 7 days of exposure, we found PS-MPs in the compound eye and ocelli of flies, which may have an adverse effect on vision ability, as seen in the water flea *Daphnia magna* after 21 days of exposure to PS-MPs^64^. Furthermore, PS-MPs were found to modulate gene expression and receptor potential amplitude in the retina of *D. melanogaster* flies at a concentration (50 µg/L) similar to that used in the current study^65^, implying long-term negative consequences.

Both male and female flies exposed to PS-MPs excreted a large number of pellets that fell uncontrollably while walking, similar to those seen in patients suffering from diarrhea or urinary incontinence. This might be because PS-MPs accumulated in various tissues, which could induce physiological and metabolic disturbances in the flies' gut, as found in previous research^66^. Although, no observable histopathological effects of PS-MPs on midgut and hind gut tissues were found, we did find concentration-dependent effects of PS-MPs on the cellular structure of midgut cells of *D. melanogaster* flies. Between columnar cells and inside the nucleus, intracellular clefts were seen, along with vacuolated cellular membrane, aberrant basement membrane folding, indentation in the nuclear membrane, matrix lysis, and rupture of mitochondrial cristae. Previous research has found comparable effects in bees that have been chronically exposed to PS-MPs^63^. The blue mussel (*Mytilus edulis* L.) also underwent considerable histological alterations after ingesting high-density polyethylene (HDPE) particles^67^. Additionally, comparable outcomes were observed in our earlier investigations, in which *D. melanogaster* flies and honey bees were repeatedly exposed however, to CdO or /and PbO NPs^68,69^. These ultrastructural changes reflect the hallmarks of cell necrosis and apoptosis^70,71^. More research is needed to investigate the potential histological and cellular changes that MPs of various shapes and sizes, either alone or in combination with other xenobiotics (metal ions, nanomaterials, and persistent organic pollutants), may induce.

## Conclusions

We revealed that flies can truly differentiate and ignore MPs-treated food. Females are more resistant to PS-MP toxicity than males, indicating that *Drosophila* has sex specific responses. PS-MPs fluorescent beads can accumulate in adult *D. melanogaster* gut tissues, Malpighian tubules, and translocate to hemolymph, body cavity, and ocelli. Seven days of exposure to PS-MPs in both male and female animals resulted in a number of cellular changes in the tissues of the midgut that are indicative of cell necrosis and apoptosis. Our research adds to our understanding of MP toxicity and will be useful in assessing the ecological risks associated with MPs as newly emerging pollutants.

## Materials and methods

### Drosophila rearing

Oregon-R wild-type *D. melanogaster* flies (#2376, Bloomington *Drosophila* stock center) were utilized in all experiments. The conditions in the rearing lab are 25 °C, 50–60% humidity, and an 18–6 h light/dark cycle^68^. Used as a food-rearing medium, cornmeal-agar was kept at a temperature of 25 °C and a relative humidity of 50–60%. It has a 14–15 g agar content, 18.5 g yeast content, 61 g glucose content, 30.5 g sucrose content, and 101 g corn meal content per liter (RH).

### Polystyrene microplastics

The latex beads were carboxylate-modified polystyrene (PS) that were fluorescent yellow-green, particle size: 2 µm, mean diameter: 1.80—2.20-micron, fluorescence: λex ~ 470 nm; λem ~ 505 nm, density: 1.04–1.05, charge density: 0.003–0.030 meq, solid content: 2.5% wt., preservative: 0% Sodium Azide, quality level: MQ100 (Sigma, Aldrich; # L4530). Polystyrene particles have been found in soils^27,72,73^. Moreover, previous research used *Drosophila* as a model for microplastic toxicity had exposed the flies to PS-MPs concentrations ranged from (0.01- 200 µg ml^−1^)^32,33,44^. We were more conservative in our study, and we used three concentrations (0.005, 0.05, and 0.5 µg/ml) that were orders of magnitude lower than those used in previous research and, presumably, those found in the environment. The working solution (25 µg ml^−1^) was prepared by dissolving 20 µl of the stock solution in 20 ml of 10% sucrose solution. PS-MPs solution was then sonicated for 30 min using an ultrasonic system (Powersonic 405) before use. Three serially diluted (10x) concentrations of PS-MPs (0.005, 0.05, 0.5 µg ml^−1^) in a standard medium were prepared for bioassays with adult *D. melanogaster*.

### Food choice assay

The food choice assay was performed according to^74^ with minor modifications. In brief, 4 h-starved male, or female flies (n = 10) were collected and placed in a petri dish with 0.5 ml of control media on one side and 0.5 ml of PS-MPs supplemented media on the other as an assay plate. Flies were free to move around and choose their preferred food before being counted every 5 min for 30 min as they stood and attempted to feed on each food. For each sex, the experiment was repeated three times.

### Food intake (CAFÉ assay)

The capillary feeder (CAFE) assay was used to investigate the potential effects of PS-MP exposure on adult food intake^75^. Simply, standard fly vials with a sponge bung on the end and 5 ml of 1% agar in them were used. Capillaries with 10 µl of sucrose solution (5% w/v) (control) or sucrose solution combined with various concentrations of PS-MPs (0.5, 0.05, or 0.005 µg/ml) were inserted into the sponge. One drop of blue food coloring was added to the sugar syrup from both the control and treated flies to ensure that the flies ingested the syrup from the capillary tubes. After being starved for four hours, flies (n = 3) were moved to CAFE champers. The same chambers were maintained without flies as evaporation controls. After 24 h, food capillaries were taken out, photographed, and ImageJ software was used to determine how much liquid food had been consumed. Then it was determined how much food was consumed by each fly in a 24-h period. The sexes were tested separately, and the experiment was repeated three times for each PS-MPs concentration.

### Survival assay

In food vials containing non-treated media or media supplemented with PS-MPs at concentrations (0.005, 0.05, and 0.5 µg PS-MPs ml^−1^), five pairs of newly hatched adult flies (5 females and 5 males) were added. To prevent generational accumulation, improve food competence, and make data collection easier, food media were changed every two days. Flies were only exposed to PS-MPs for 7 days before being moved to untreated media. The number of dead flies of both sexes was then recorded daily for 20 days in order to investigate the long-term effect of a short-term exposure to different PS-MP concentrations on fly survival. The experiment was carried out three times.

### Starvation resistance assay

The capacity of an organism to withstand starvation may indicate its fitness. Therefore, starvation resistance is a commonly measured trait in in *Drosophila* studies^61,76^. So, we investigated whether exposure to PS-MPs might alter the ability of both male and female flies to withstand starvation. To achieve this, newly emerged adults were exposed for 7 days to either a controls or various PS-MPs supplemented food media concentrations. Seven adult fly pairs were then moved to petri dishes devoid of food media. Dead flies were noted after 24 h. There were three duplicates of the experiment. This experiment was carried out in accordance with^77^, with minor modifications.

### Fecal pellet count and integrated intensity, and PS-MPs accumulation

For 7 days, groups of 2 h starved adult flies (male and female) (n = 5) were fed blue food media with either control or PS-MPs supplementation. The flies were then placed in Petri dishes. A light microscope was used to count all 24 h-fecal pellets for both control and PS-MPs exposed flies. Fecal pellets from adults who had been exposed to PS-MPs were then photographed using an Olympus BX53 digital fluorescence microscope. The amount of PS-MPs excreted in fecal pellets was measured by determining the integrated fluorescence intensity using ImageJ software. Both sexes' feces were examined separately. The experiment was repeated 3 times.

After being fed either control or PS-MPs supplemented food media for 7 days, adult flies were dissected to detect the presence and distribution of PS-MPs in the digestive tract, fat body, and/or Malpighian tubules. After mounting the refereed tissues on a microscope slide, they were examined with an Olympus BX53 digital fluorescence microscope. The sexes were studied separately.

### Effects on cellular structure (TEM)

Utilizing an electron transmission microscope JEM-1200EX (JEOL, Japan) at an accelerating voltage of 60 kV, midguts from three composite samples (n = 5) of PS-MPs-treated and untreated male and female flies were studied, and photographed (for more information, see^69^.

### Statistical analysis

Data were analyzed using GraphPad Prism version 8.00 for Windows (www.graphpad.com). Normality of data was assessed by use of the Kolomogrov- Smirnov test, and homogeneity of variance was assessed by use of Levene's test. The impact of treatments on survival of flies has been assessed by logrank (Mantel cox) paired test, *p* < 0.05 after Bonferroni correction (i.e. a conservative test that protects from Type 1 Error to counteract the problem of multiple comparisons). Differences in food choice were assessed by Student’s t-test. Effect of treatment on food intake, starvation resistance and fecal pellets count were assessed by one-way ANOVA followed by Tukey’s post hoc test. An alpha level of 0.05 was used for all tests.

## Supplementary Information

Supplementary Information.

## Data Availability

The datasets generated during and/or analysed during the current study are available from the corresponding author on reasonable request.
