# Supplementary material for: Exposure to polystyrene microplastic beads causes sex-specific toxic effects in the model insect Drosophila melanogaster
Source: Sci Rep. 2023 Jan 5;13:204. doi: 10.1038/s41598-022-27284-7 (PMC9814852; doi:10.1038/s41598-022-27284-7)
Supplement: Supplementary file 1 — Supplementary Information. [file 41598_2022_27284_MOESM1_ESM.doc]

**Exposure to Polystyrene Microplastic Beads Causes Sex-Specific Toxic Effects in the Model Insect *Drosophila melanogaster***

Samar El Kholy^*^ & Yahya Al Naggar

Zoology Department, Faculty of Science, Tanta University, 31527 Tanta, Egypt

Corresponding:

samar_elkholy@science.tanta.edu.eg (S. El Kholy)


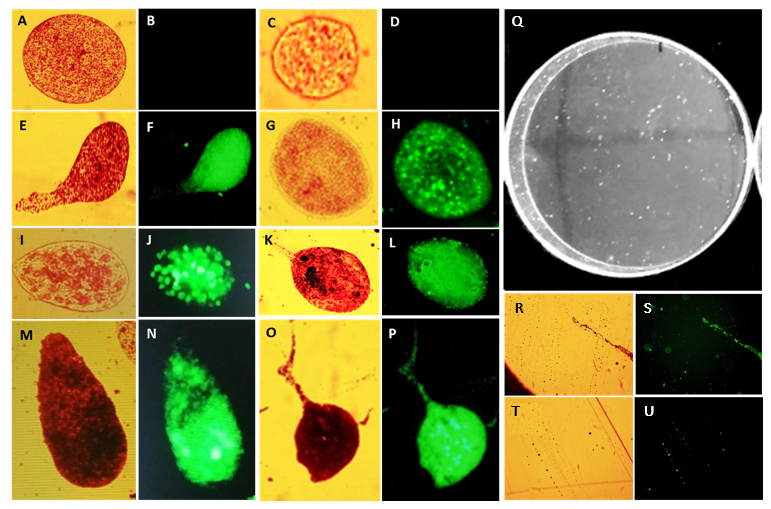


**Figure S1**. Fecal pellets of adult *D. melanogaster flies* exposed to green-yellow, fluorescent polystyrene microplastics (PS-MPs). A-B are female’s fecal pellets of control, and E-F, I-J, and M-N are fecal pellets of female flies exposed to 0.005, 0.05 and 0.5 µg/ml PS-MPs, respectively. C-D are male’s fecal pellets of control, and G-H, K-L and O-P are fecal pellets of flies exposed 0.005, 0.05 and 0.5 µg/ml, respectively. Q, the green-yellow fluorescent of PS-MPs appeared in the fecal pellets of PS-MPs treated flies; Petri dish was photographed on UV plate. R-S, showing excreta of female, and T-U showing excreta of male flies exposed to 0.005 µg/ml PS-MPs. Fecal pellets appear smaller than usual, as if falling uncontrollably while walking, similar to those seen in diarrhea or urinary incontinence patients.
